# Safety and tolerability of eptinezumab in patients with migraine: a pooled analysis of 5 clinical trials

**DOI:** 10.1186/s10194-021-01227-5

**Published:** 2021-03-30

**Authors:** Timothy R. Smith, Egilius L. H. Spierings, Roger Cady, Joe Hirman, Barbara Schaeffler, Vivienne Shen, Bjørn Sperling, Thomas Brevig, Mette Krog Josiassen, Elizabeth Brunner, Loan Honeywell, Lahar Mehta

**Affiliations:** 1 StudyMetrix Research, LLC, 3862 Mexico Road, St. Peters, MO 63303 USA; 2 Medvadis Research Corporation, Boston PainCare, Waltham, MA USA; 3 Lundbeck La Jolla Research Center, San Diego, CA USA; 4 Pacific Northwest Statistical Consulting, Inc., Woodinville, WA USA; 5 Lundbeck Seattle BioPharmaceuticals, Inc., Seattle, WA USA; 6 Lundbeck LLC, Deerfield, IL USA; 7 H. Lundbeck A/S, Copenhagen, Denmark

**Keywords:** Eptinezumab, Episodic migraine, Chronic migraine, Safety, Tolerability

## Abstract

**Background:**

The humanized anti-CGRP monoclonal antibody eptinezumab has been evaluated in five large-scale clinical trials conducted in patients with migraine. This integrated analysis was conducted to evaluate the comprehensive safety and tolerability of eptinezumab in patients with migraine across these studies.

**Methods:**

Data were pooled from four randomized, double-blind, placebo-controlled studies and the first year of one open-label study.

**Results:**

The pooled population comprised 2867 adults with migraine: eptinezumab, *n* = 2076 (4797 infusions); placebo, *n* = 791 (1675 infusions). A total of 1137/2076 (54.8%) patients who received eptinezumab and 414/791 (52.3%) patients who received placebo experienced ≥1 treatment-emergent adverse event (TEAE); rates were similar across eptinezumab dose groups (10–1000 mg). For most patients with TEAEs, the events were mild or moderate in severity and considered unrelated to study drug by the investigators. Thirty infusion-site AEs occurred in 27/2076 (1.3%) patients who received eptinezumab and 7 in 7/791 (0.9%) patients who received placebo. Infusion-site AEs led to infusion interruption in 19/2076 (0.9%) and 5/791 (0.6%) patients in the eptinezumab and placebo groups, respectively. Nasopharyngitis occurred in ≥2% of patients in the eptinezumab 300-mg group and with an incidence of at least 2 percentage points greater than in the placebo group; however, in most patients (eptinezumab, 139/140; placebo 40/41), its occurrence was considered not related to study treatment. Adverse events coded to hypersensitivity occurred for 23/2076 (1.1%) patients treated with eptinezumab and no patients in the placebo group. If additional TEAE terms that could indicate hypersensitivity are considered (e.g., urticaria, flushing/hot flush, rash, and pruritus), hypersensitivity reactions in the two pivotal placebo-controlled phase 3 studies occurred in ≥2% of patients in the eptinezumab 100-mg and 300-mg groups, and the incidence was at least 2 percentage points greater in either of these groups than in the placebo group. Most hypersensitivity reactions were not serious and resolved with standard medical treatment or observation without treatment, usually within 1 day.

**Conclusions:**

In adults with migraine, the intravenous administration of eptinezumab every 12 weeks demonstrated a favorable safety and tolerability profile.

**Trial registration:**

ClinicalTrials.gov (Identifiers: NCT01772524, NCT02275117, NCT02559895, NCT02974153, NCT02985398).

**Supplementary Information:**

The online version contains supplementary material available at 10.1186/s10194-021-01227-5.

## Introduction

Migraine is a highly prevalent paroxysmal neurological disorder that is characterized by recurrent episodes of moderate to severe headache associated with physiological disruptions of neurological, gastrointestinal, and sensory function, as well as mood changes [1]. Globally, migraine is the leading cause of disability for people below the age of 50 years [2], with the extent of disability dependent upon the frequency of attacks (2 or more per month in 42% to 50% of patients), the duration of the attack (more than 24 h in 39% of patients), the intensity of the attack (severe or very severe in 48% to 74% of patients), the accompanying symptoms, and alterations in professional, social, and familial quality of life [3].

The primary goals of effective preventive migraine treatment are to decrease the frequency and severity of migraine, reduce the reliance on acute medication, prevent migraine attacks from becoming more frequent, and reduce migraine-related impact, thereby improving overall quality of life [4]. A number of antiepileptics, beta-blockers, antidepressants, and calcium channel antagonists have demonstrated at least some level of efficacy and are commonly prescribed for use [5]. Side effects—particularly adverse gastrointestinal, cognitive, cardiovascular, and renal events with oral preventive medications—may limit tolerability in some patients. In contrast, the calcitonin gene-related peptide (CGRP) monoclonal antibodies have demonstrated improvements in monthly migraine days with an adverse event profile similar to placebo. In one recent (2020) meta-analysis of CGRP monoclonal antibodies in episodic migraine (EM), only injection-site pain was reported significantly more often with active treatment than with placebo [6]; however, United States prescribing labels indicate that hypersensitivity [7–10] and constipation [7] are adverse events to surveil in patients treated with CGRP monoclonal antibodies. OnabotulinumtoxinA treatment, approved for the preventive treatment of chronic migraine (CM), is generally well tolerated, though is associated with elevated rates of blepharoptosis, muscle weakness, neck pain, and injection-site pain [11].

Eptinezumab is a humanized CGRP monoclonal antibody indicated for the preventive treatment of migraine in adults [8]. Eptinezumab has been evaluated in five large-scale clinical trials conducted in patients with migraine, encompassing four randomized double-blind, placebo-controlled, parallel-group studies [12–15] and one open-label, long-term safety study [16]. In the two pivotal phase 3 PROMISE studies, eptinezumab 100 mg or 300 mg was administered by intravenous (IV) infusion every 12 weeks and demonstrated a statistically significant reduction from baseline in the frequency of migraine headache days during weeks 1 to 12 compared with placebo that was sustained or further improved with additional dosing [14, 15]. This integrated analysis was conducted to evaluate the comprehensive safety and tolerability of eptinezumab across the broad spectrum of patients with migraine, utilizing trial data collected throughout the eptinezumab clinical development program.

## Methods

Source studies are summarized in Table 1. Four of the five studies were placebo-controlled (NCT01772524, NCT02275117, PROMISE-1, and PROMISE-2), and the other was open-label (PREVAIL). In all five trials, the study drug (eptinezumab 10 mg, 30 mg, 100 mg, 300 mg, 1000 mg, or placebo) was administered by IV infusion. Two trials (NCT01772524 and NCT02275117) were single-dose studies, and the other three (PROMISE-1, PROMISE-2, and PREVAIL) were multi-dose studies in which study medication was administered once every 12 weeks. PREVAIL was ongoing at the time of this analysis. It comprised two treatment phases: a primary treatment phase that included four eptinezumab infusions administered 12 weeks apart and a secondary treatment phase that included four additional infusions administered 12 weeks apart. Only data through the primary treatment phase (week 48) were included in the integrated safety database. The integrated analysis utilized the safety populations of these studies, which comprised patients who received at least 1 dose of study drug. Data were summarized descriptively by dose and across doses for patients with EM and CM combined. Patients were summarized by treatment group.

**Table 1:** Eptinezumab clinical studies conducted in patients with migraine

Study	Phase	Number Treated	Study Design	Study Drugs/Doses	Dosing Frequency	Scheduled Post-Dose Visits
NCT01772524 [12]	1b	163 (EM)	Double-blind, randomized, placebo-controlled, parallel group	Eptinezumab 1000 mg or placebo	Single dose (day 0)	Week 2 and months 1, 2, 3, and 6
NCT02275117 [13]	2	616 (CM)	Double-blind, randomized, placebo-controlled, parallel group	Eptinezumab 10 mg, 30 mg, 100 mg, or 300 mg, or placebo	Single dose (day 0)	Months 1, 2, 3, 6, and 9, and week 49
PROMISE-1 (NCT02559895) [14]	3	888 (EM)	Double-blind, randomized, placebo-controlled, parallel group	Eptinezumab 30 mg, 100 mg, or 300 mg, or placebo	Day 0 and every 12 weeks through week 36 (4 doses)	Months 1, 2, 3, 4, 5, 6, 7, 9, 12, and 14
PROMISE-2 (NCT02974153) [15]	3	1072 (CM)	Double-blind, randomized, placebo-controlled, parallel group	Eptinezumab 100 mg or 300 mg or placebo	Day 0 and week 12 (2 doses)	Week 2 and months 1, 2, 3, 4, 5, 6, and 8
PREVAIL (NCT02985398) [16]	3	128 (CM)	Open-label, uncontrolled	Eptinezumab 300 mg	Day 0 and every 12 weeks through week 84 (8 doses)^a^	Week 2 and months 1, 2, 3, 6, 9, 12, 15, 18, 21, and 26

*CM* chronic migraine, *EM* episodic migraine

^a^PREVAIL was ongoing at the time of this analysis; only data through the primary treatment phase (week 48) were included in the integrated safety database

Inclusion and exclusion criteria for the individual studies have been published [12–16], were generally similar across studies, and will be briefly summarized here. Across studies, eligible patients included adults with EM or CM (International Classification of Headache Disorders criteria) and a history of migraine for ≥12 months prior to screening. Individuals were excluded if they had confounding pain syndromes or any pain syndrome requiring regular analgesia; uncontrolled or untreated psychiatric conditions; temporomandibular disorders; or present or previous malignancies. Also excluded were patients who received any monoclonal antibody treatment or botulinum toxin treatment within 3–6 months of screening. Patients using barbiturates or prescription opioids for at least 4 days per month were eligible for participation if their use was stable for at least 2 months before screening, with this restriction maintained throughout the treatment period.

Medical history and delineation of some cardiovascular risk factors were obtained through patient medical records or from patient interviews (if records were not available). Medical history was coded using the Medical Dictionary for Regulatory Activities (MedDRA) version 20.1 for PROMISE-1, PROMISE-2, and PREVAIL; MedDRA version 15.0 was used for NCT01772524 and NCT02275117. Captured cardiovascular risk factors included medical history of hypertension-, hyperlipidemia-, or diabetes-related conditions; history of ischemic cardiovascular events or procedures; obesity (body mass index [BMI] ≥30 kg/m^2^); male ≥45 years of age or female ≥55 years of age; and black or African American race.

Safety assessments included the evaluation of treatment-emergent adverse events (TEAEs); the incidence, nature, and severity of TEAEs; clinical laboratory tests, serum anti-drug antibody testing, and vital sign assessments; 12-lead electrocardiograms; and the Columbia Suicide Severity Rating Scale. Verbatim descriptions of TEAEs recorded by investigators were mapped to the MedDRA thesaurus terms and converted to the same version (version 20.1) for all studies.

For all five studies, each TEAE was assessed by the investigator with regard to (1) seriousness, (2) severity, and (3) relationship to study drug. Also summarized were TEAEs leading to study-drug discontinuation or infusion interruption. TEAEs were considered serious by the investigator or sponsor if they (1) resulted in death, (2) were life-threatening, (3) led to hospitalization or prolonged hospitalization, (4) led to persistent or significant incapacity or substantial disruption of the ability to conduct normal life functions, (5) resulted in congenital anomaly or birth defect, or (6) were considered to be an important medical event by the investigator (i.e., may not have resulted in death, were not life-threatening, or did not require hospitalization, but may have jeopardized the patient and/or required medical or surgical intervention to prevent an outcome listed in the definition). Severity was graded on a 5-point scale: 1 = mild, 2 = moderate, 3 = severe, 4 = life-threatening, 5 = death. The medical judgment of the investigator was used to determine the likely relationship of the TEAE to the study drug, considering all relevant factors, including (but not limited to) medical history, concomitant medical conditions, and concomitant medications. Determination was based on the assessment of temporal relationships, biologic plausibility, association with underlying disease, and presence of a more likely cause. For studies NCT01772524, NCT02275117, PROMISE-1, and PROMISE-2, any event with a start date and time on or after the date and time of first administration of study medication was considered a TEAE. For PREVAIL, any event with a start date and time on or after the date and time of the first dose of study drug until the end of the primary treatment phase (week 48) was considered a TEAE.

Approval for each study was provided by the independent ethics committee or institutional review board of the study sites. All studies were conducted in accordance with Good Clinical Practice guidelines, the principles of the Declaration of Helsinki, and all applicable regulatory requirements. Patients provided written informed consent prior to initiation of any study procedures.

## Results

### Patients

**Fig. 1:**
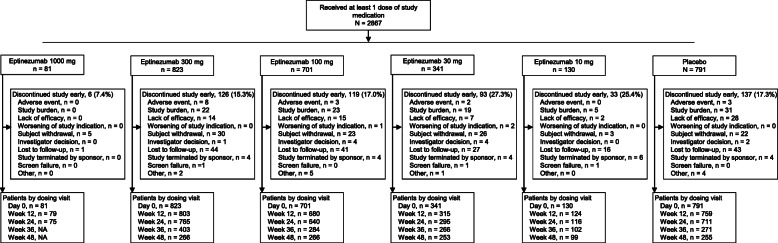
Patient Disposition. NA, not applicable

The integrated population comprised 2867 patients with migraine (EM, *n* = 1051; CM, *n* = 1816) who received at least 1 dose of eptinezumab (*n* = 2076) or placebo (*n* = 791). Among those who received eptinezumab, 130 patients received 10 mg (single dose), 341 patients received 30 mg (up to 4 doses), 701 patients received 100 mg (up to 4 doses), 823 patients received 300 mg (up to 4 doses), and 81 patients received 1000 mg (single dose). Most patients completed the assigned study drug regimen (91%) and completed study participation (82%; Fig. 1).

Demographic and baseline characteristics for the overall integrated safety population are summarized in Table 2. The mean age of the total population (eptinezumab + placebo) was 39 years; patients were predominantly female (86%) and white (87%).

**Table 2:** Demographic and baseline characteristics of the pooled safety population

	Eptinezumab						Placebo
	1000 mg	300 mg	100 mg	30 mg	10 mg	All	
N	81	823	701	341	130	2076	791
Population	EM	EM + CM	EM + CM	EM + CM	CM	EM + CM	EM + CM
Mean (SD) age, years	38.6 (10.8)	40.3 (10.9)	39.9 (11.1)	37.9 (10.9)	36.4 (10.3)	39.5 (11.0)	39.3 (11.0)
Mean (SD) BMI, kg/m^2^	27.5 (5.2)	27.4 (5.8)	27.6 (6.1)	28.9 (7.6)	27.4 (5.4)	27.7 (6.2)	27.9 (6.1)
Sex, % female	82.7	87.5	84.2	86.8	86.9	86.0	86.7
Race, %^a^							
White	81.5	90.5	90.7	83.0	86.9	88.8	85.6
Black/African American	12.3	7.0	7.1	13.2	9.2	8.4	10.6
Asian	4.9	0.5	0.3	0.3	0.8	0.6	0.9
American Indian/Alaska Native	0	0.4	0.1	0.3	1.5	0.3	0.4
Native Hawaiian/Pacific Islander	0	0.2	0.1	0.3	0	0.2	0.3
Multiple races	1.2	1.1	1.3	1.5	0	1.2	1.6
Other	0	0.1	0.3	1.5	1.5	0.5	0.6
Not reported	0	0.1	0	0	0	< 1	0

*BMI* body mass index, *CM* chronic migraine, *EM* episodic migraine, *SD* standard deviation

^a^Due to rounding, the sum of percentages may not equal 100

The most frequently reported medical history conditions generally were well balanced across treatment groups and included seasonal allergy (20.2%), drug hypersensitivity (16.8%), anxiety (14.5%), depression (13.7%), and insomnia (12.5%). Positive medical histories for conditions related to hypertension, hyperlipidemia, or diabetes (glucose tolerance impaired, hyperglycemia, or impaired fasting glucose) were infrequent due to study exclusion criteria related to these conditions (3.5%, 6.1%, and < 1%, respectively, at baseline). Nearly one-third (32.4%) of all patients were classified as obese (BMI ≥30 kg/m^2^), and nearly half (48.9%) had at least 1 cardiovascular risk factor at baseline.

Across the five studies, most patients (98.2%) were taking at least 1 concomitant medication at baseline, the most common of which were ibuprofen (38.2%), aspirin/acetaminophen/caffeine combination (31.9%), sumatriptan (29.4%), and paracetamol (17.2%). Approximately 14.2% of patients were taking at least 1 cardiovascular medication at baseline, with use being similar among the eptinezumab 300-mg (17.3%), eptinezumab 100-mg (15.3%), and placebo (14.0%) groups, and lower in the 1000-mg (4.9%), 30-mg (9.4%), and 10-mg (9.2%) eptinezumab groups. The most commonly used cardiovascular medications at baseline (≥1% of the combined eptinezumab or placebo groups) were propranolol (2.4%), metoprolol (2.2%), and bisoprolol (0.6%), and it is probable that these medications had been prescribed for migraine rather than for cardiovascular indications.

### Exposure

Overall, 4797 eptinezumab infusions were administered to 2076 patients in these studies. A total of 1334 patients received at least 6 months of eptinezumab treatment (2 doses approximately 12 weeks apart plus follow-up for at least 5 half-lives [130 days]), including 637 who received eptinezumab 300 mg and 511 who received eptinezumab 100 mg. Among the eptinezumab groups, 490 patients had 1 year of treatment (4 doses approximately 12 weeks apart plus follow-up for at least 5 half-lives), including 167 patients who received eptinezumab 300 mg and 171 who received eptinezumab 100 mg.

### Adverse events

Rates of TEAEs are summarized in Table 3. A total of 1137/2076 (54.8%) patients who received eptinezumab and 414/791 (52.3%) patients who received placebo experienced 1 or more TEAEs during studies NCT01772524, NCT02275117, PROMISE-1, PROMISE-2, and PREVAIL (primary treatment phase only). TEAE rates were similar across eptinezumab dose groups: 56.8%, 56.7%, 52.2%, 54.0%, and 56.9% of patients who received eptinezumab 1000 mg, 300 mg, 100 mg, 30 mg, or 10 mg, respectively, experienced at least 1 TEAE.

**Table 3:** Summary of treatment-emergent adverse events (TEAEs) for the pooled safety population

	Eptinezumab						Placebo
*Patients, n (%)*	1000 mg^**a**^ (***n*** = 81)	300 mg^b^ (***n*** = 823)	100 mg^b^ (***n*** = 701)	30 mg^b^ (***n*** = 341)	10 mg^**c**^ (***n*** = 130)	All (***N*** = 2076)	(***N*** = 791)
Any TEAE	46 (56.8)	467 (56.7)	366 (52.2)	184 (54.0)	74 (56.9)	1137 (54.8)	414 (52.3)
Any study-drug–related TEAE^d^	16 (19.8)	124 (15.1)	92 (13.1)	42 (12.3)	21 (16.2)	295 (14.2)	74 (9.4)
Any serious TEAE	2 (2.5)	17 (2.1)	11 (1.6)	4 (1.2)	1 (0.8)	35 (1.7)	11 (1.4)
Any TEAE leading to study drug discontinuation	0	19 (2.3)	9 (1.3)	12 (3.5)	0	40 (1.9)	8 (1.0)
Any TEAE leading to interruption of study-drug infusion	0	19 (2.3)	11 (1.6)	10 (2.9)	0	40 (1.9)	6 (0.8)

^a^ Single dose study with follow-up for 24 weeks (NCT01772524)

^b^ Single- and multiple-dose studies (NCT02275117, PROMISE-1, PROMISE-2, and PREVAIL) with follow-up ranging from 32 to 56 weeks

^c^ Single-dose study with follow-up for 49 weeks (NCT02275117)

^d^ Relatedness determined by investigator

TEAEs occurring in at least 2% of any eptinezumab treatment group, with an incidence of at least 2 percentage points greater than in the placebo group, are summarized in Table 4. There was no apparent association between the dose of eptinezumab and the incidence of these TEAEs; the events tended to occur after the first dose, with the incidence decreasing after subsequent doses.

**Table 4:** Summary of treatment-emergent adverse events (TEAEs) with incidence of ≥2% of patients in any eptinezumab arm and 2 percentage points greater than placebo

	Eptinezumab						Placebo
*Patients, n (%)*	1000 mg (***n*** = 81)	300 mg (***n*** = 823)	100 mg (***n*** = 701)	30 mg (***n*** = 341)	10 mg (***n*** = 130)	All (***N*** = 2076)	(***N*** = 791)
Upper respiratory tract infection	7 (8.6)	64 (7.8)	45 (6.4)	32 (9.4)	9 (6.9)	157 (7.6)	48 (6.1)
Nasopharyngitis	1 (1.2)	72 (8.7)	44 (6.3)	17 (5.0)	6 (4.6)	140 (6.7)	41 (5.2)
Dizziness	3 (3.7)	16 (1.9)	27 (3.9)	11 (3.2)	11 (8.5)	68 (3.3)	21 (2.7)
Fatigue	3 (3.7)	24 (2.9)	20 (2.9)	9 (2.6)	2 (1.5)	58 (2.8)	13 (1.6)
Anxiety	0	14 (1.7)	10 (1.4)	5 (1.5)	4 (3.1)	33 (1.6)	5 (0.6)
Pain in extremity	1 (1.2)	6 (0.7)	5 (0.7)	8 (2.3)	0	20 (1.0)	2 (0.3)
Tooth abscess	3 (3.7)	4 (0.5)	3 (0.4)	2 (0.6)	1 (0.8)	13 (0.6)	3 (0.4)
Dry mouth	3 (3.7)	3 (0.4)	2 (0.3)	1 (0.3)	1 (0.8)	10 (0.5)	3 (0.4)
Sciatica	2 (2.5)	2 (0.2)	5 (0.7)	1 (0.3)	0	10 (0.5)	2 (0.3)
Fall	1 (1.2)	3 (0.4)	2 (0.3)	0	3 (2.3)	9 (0.4)	2 (0.3)
Arthropod bite	2 (2.5)	3 (0.4)	1 (0.1)	1 (0.3)	0	7 (0.3)	4 (0.5)
Weight decreased	2 (2.5)	2 (0.2)	1 (0.1)	0	1 (0.8)	6 (0.3)	1 (0.1)
Electrocardiogram QT prolonged	3 (3.7)	0	0	0	0	3 (0.1)	1 (0.1)

The majority of TEAEs were considered by the investigator as not related to study drug. A total of 295/2076 (14.2%) patients who received eptinezumab and 74/791 (9.4%) patients who received placebo experienced 1 or more treatment-related AE (related TEAE). The only related TEAE that occurred in at least 2% of the overall eptinezumab population was fatigue (41/2076 [2.0%]), which also was reported for 7/791 [0.9%] patients who received placebo. Related TEAEs in the class of gastrointestinal disorders occurred in 62/2076 (3.0%) patients who received eptinezumab and 18/791 (2.3%) patients who received placebo; however, no single event in this class was observed in at least 2% of the overall eptinezumab population. Nausea was the most common gastrointestinal-related TEAE (eptinezumab, 39/2076 [1.9%]; placebo, 8/791 [1.0%]). Other gastrointestinal-related TEAEs that occurred in ≥2 eptinezumab-treated patients were vomiting (eptinezumab, 11/2076 [0.5%]; placebo, 3/791 [0.4%]), constipation (eptinezumab, 7/2076 [0.3%]; placebo, 1/791 [0.1%]), dry mouth (eptinezumab, 6/2076 [0.3%]; placebo, 2/791 [0.3%]), diarrhea (eptinezumab, 5/2076 [0.2%]; placebo, 4/791 [0.5%]), and dyspepsia (eptinezumab, 2/2076 [< 0.1%]; placebo, 0/791 [0%]).

For the majority of patients with TEAEs, the events were classified as mild or moderate in severity. A total of 54/2976 (2.6%) patients who received eptinezumab and 19/791 (2.4%) patients who received placebo experienced 1 or more severe events (grade 3 or higher). Of these, 6 events that occurred in 4 patients (eptinezumab, *n* = 3; placebo, *n* = 1) were considered related to study treatment (Supplemental Table 1). These included nausea (eptinezumab 1000 mg, *n* = 1), vomiting (eptinezumab 1000 mg, *n* = 1), migraine (placebo, *n* = 1), meralgia paresthetica (eptinezumab 300 mg, *n* = 1), migraine with aura (eptinezumab 300 mg, *n* = 1), and major depression (eptinezumab 30 mg, *n* = 1). No patient who received eptinezumab had a life-threatening (grade 4) or fatal (grade 5) TEAE. One patient in the placebo group experienced two grade 4 events (apnea and chronic obstructive pulmonary disease); both were considered not related to study treatment.

A total of 40/2076 (1.9%) patients who received eptinezumab and 8/791 (1.0%) patients who received placebo had a TEAE that led to study-drug discontinuation, half of which were deemed related to study drug (Table 5). Events coded to hypersensitivity led to discontinuation of study treatment in 15 (0.7%) patients who received eptinezumab and in no patient who received placebo. In accordance with study protocols, a conservative medical approach generally was taken for these patients: there was no rechallenge of the study drug after its discontinuation. Hypertension led to study-drug discontinuation in 2 (< 0.1%) patients who received eptinezumab (100 mg, *n* = 1; 30 mg, *n* = 1) and in no patient who received placebo. All other TEAEs leading to discontinuation each occurred in 1 patient who received eptinezumab and/or 1 patient who received placebo.

**Table 5:** Summary of treatment-emergent adverse events (TEAEs) leading to discontinuation that were considered related to study drug

	Eptinezumab						Placebo
*Patients, n (%)*	1000 mg (***n*** = 81)	300 mg (***n*** = 823)	100 mg (***n*** = 701)	30 mg (***n*** = 341)	10 mg (***n*** = 130)	All (***N*** = 2076)	(***N*** = 791)
Hypersensitivity	0	10 (1.2)	1 (0.1)	4 (1.2)	0	15 (0.7)	0
Alopecia	0	0	0	0	0	0	1 (0.1)
Peripheral swelling	0	0	0	1 (0.3)	0	1 (< 0.1)	0
Blood pressure increased	0	0	1 (0.1)	0	0	1 (< 0.1)	0
Hypertension	0	0	1 (0.1)	0	0	1 (< 0.1)	0
Dermatitis bullous	0	0	1 (0.1)	0	0	1 (< 0.1)	0
Headache (worsening of)	0	1 (0.1)	0	0	0	1 (< 0.1)	0
Erythema	0	0	1 (0.1)	0	0	1 (< 0.1)	0
Infusion-site erythema	0	1 (0.1)	0	0	0	1 (< 0.1)	0
Anaphylactic reaction	0	1 (0.1)	0	0	0	1 (< 0.1)	0

Relationship of TEAE to study drug was determined by the investigator

A total of 40/2076 (1.9%) patients in the eptinezumab population and 6/791 (0.8%) patients in the placebo population had a TEAE that led to interruption of study-drug infusion. The interruption-causing events that occurred in more than 1 patient in either overall study arm included hypersensitivity (eptinezumab, 16/2076 [0.8%]; placebo, 0/791 [0%]), infusion-site extravasation (eptinezumab, 15/2076 [0.7%]; placebo, 6/791 [0.8%]), infusion-site pain (eptinezumab, 4/2076 [0.2%]; placebo, 0/791 [0%]), and nausea (eptinezumab, 2/2076 [< 0.1%]; placebo, 0/791 [0%]). The following events led to interruption of study-drug infusion in 1 patient each: anaphylactic reaction (eptinezumab 300 mg), asthma (eptinezumab 100 mg), feeling cold (eptinezumab 300 mg), infusion-site discomfort (eptinezumab 100 mg), infusion-site pruritus (eptinezumab 30 mg), rhinitis (eptinezumab 300 mg), and throat irritation (eptinezumab 100 mg). All events leading to infusion interruption were mild or moderate in severity, lasted 1 day or less, and resolved spontaneously or through event-specific treatment.

#### Serious adverse events

Serious AEs (SAEs) occurred infrequently (eptinezumab, 35/2076 [1.7%]; placebo, 11/791 [1.4%]). Five SAEs occurred in more than 1 patient: uterine leiomyoma (eptinezumab 300 mg, *n* = 2; eptinezumab 100 mg, *n* = 1), seizure (eptinezumab 300 mg, *n* = 2), suicide attempt (eptinezumab 300 mg, n = 1; eptinezumab 100 mg, n = 1), suicidal ideation (eptinezumab 100 mg, *n* = 1; eptinezumab 10 mg, *n* = 1), syncope (eptinezumab 100 mg, *n* = 1; placebo, *n* = 2), and conversion disorder (eptinezumab 1000 mg, *n* = 1; eptinezumab 300 mg, *n* = 1). None of these events was considered related to treatment.

Two patients experienced an SAE that was deemed related to study treatment. One of these patients had what appeared to be an anaphylactic reaction while receiving eptinezumab 300 mg. The event was of moderate severity (grade 2) and associated with features of an immediate type 1 (immunoglobulin E–mediated) hypersensitivity event. The patient had no clinical manifestations of respiratory or cardiovascular compromise. Therefore, upon medical review, the event was found to be inconsistent with the diagnosis of anaphylactic reaction and instead was designated as an allergic reaction and infusion reaction under the broader classification of immune system disorders [16]. The event was initially treated with epinephrine via injection, but there was no discernible effect within 10 min. The principal investigator then administered IV diphenhydramine, which resulted in an almost immediately detectable response, including reductions in itching, nasal congestion, and flushing. The other patient experienced an SAE of migraine with aura while receiving eptinezumab 300 mg. The event was described as “worsening of migrainous visual phenomena,” and the patient had a well-documented history of similar aura symptoms. The event lasted 4 days and was resolved through medication and hospitalization.

#### TEAEs related to infusion site

Thirty TEAEs related to infusion site occurred in 27/2076 (1.3%) patients who received eptinezumab, and 7 such events occurred in 7/791 (0.9%) patients who received placebo. These events led to infusion interruption in 19/2076 (0.9%) and 5/791 (0.6%) patients in the eptinezumab and placebo arms, respectively.

Infusion-site erythema led to discontinuation of the study drug in 1 patient (eptinezumab 300 mg). Extravasation was the most frequently observed infusion-site TEAE, occurring in 16/2076 (0.8%) and 5/791 (0.6%) patients in the eptinezumab and placebo populations, respectively. In all patients who experienced it, the extravasation was mild (eptinezumab, *n* = 13/16; placebo, *n* = 6/7) or moderate (eptinezumab, *n* = 3/16; placebo, *n* = 1/7) in severity and resolved within 1 day.

Other TEAEs related to infusion site were infrequent, including nerve damage (left foot neuropathy; eptinezumab, 1/2076 [< 0.1%]; placebo, 0/791 [0%]), rash (eptinezumab, 3/2076 [0.1%]; placebo, 0/791 [0%]), discomfort (eptinezumab, 1/2076 [< 0.1%]; placebo, 0/791 [0%]), eczema (eptinezumab, 1/2076 [< 0.1%]; placebo, 0/791 [0%]), erythema (eptinezumab, 1/2076 [< 0.1%]; placebo, 1/791 [0.1%]), pain (eptinezumab, 4/2076 [0.2%]; placebo, 1/791 [0.1%]), pruritus (eptinezumab, 2/2076 [< 0.1%]; placebo, 0/791 [0%]), and paresthesia (eptinezumab, 1/2076 [< 0.1%]; placebo, 0/791 [0%]). No severe (≥ grade 3) or serious infusion-site TEAE was reported.

#### Nasopharyngitis

Nasopharyngitis occurred in 140/2076 (6.7%) patients who received eptinezumab and 41/791 (5.2%) patients who received placebo. In most patients (eptinezumab, 139/140; placebo, 40/41), its occurrence was considered by the investigator as not related to study treatment. For the 2 patients who experienced nasopharyngitis that was considered related to treatment (1 with placebo; 1 with eptinezumab 100 mg), the event was described as “common cold,” lasted 5 to 7 days, was moderate in severity, and resolved with medication. Occurrences of nasopharyngitis were typically mild or moderate in severity; a single patient in the eptinezumab 300-mg group experienced severe nasopharyngitis. This suggests no relationship between eptinezumab concentration and the nasopharyngitis incidence. The incidence of nasopharyngitis was greatest following the first dose of study drug (eptinezumab, 65/2076 [3.1%]; placebo, 19/791 [2.4%]) and was lower following subsequent administration (eptinezumab, 39/2007 [1.9%], 33/1861 [1.8%], and 20/1068 [1.9%] following the second, third, and fourth doses, respectively; placebo, 16/765 [2.1%], 11/682 [1.6%], and 3/277 [1.1%] following the second, third, and fourth doses, respectively). Of the nasopharyngitis events that arose within 30 days of any dose, more occurred 15 to 30 days after dosing (*n* = 30) than on the infusion day or between 1 and 7 days after dosing (*n* = 8).

#### Cardiovascular TEAEs

Cardiac and vascular TEAEs were infrequent, occurring in 27/2076 (1.3%) and 32/2076 (1.5%) patients who received eptinezumab, respectively, and in 8/791 (1.0%) patients who received placebo, each.

The most frequently reported cardiac disorders were palpitations, which occurred in similar proportions of the overall eptinezumab and placebo arms (8/2076 [0.4%] and 3/791 [0.4%], respectively), and tachycardia (7/2076 [0.3%] and 1/791 [0.1%], respectively). Only 3 of these palpitation events (eptinezumab 10 mg, *n* = 1; placebo, *n* = 2) and none of the tachycardia events were considered related to the study drug. One patient with palpitations discontinued treatment due to the event.

Hypertension occurred in 11/2076 (0.5%) patients who received eptinezumab and 6/791 (0.8%) patients who received placebo. “Blood pressure increased” was noted for 39/2076 (1.9%) patients who received eptinezumab and 14/791 (1.8%) patients who received placebo, and “blood pressure systolic increased” was observed for 2/2076 (< 0.1%) patients who received eptinezumab and for none who received placebo. Treatment-emergent hypertension events were all mild (eptinezumab, *n* = 0/2076; placebo, *n* = 3/791) or moderate (eptinezumab, *n* = 11/2076; placebo, *n* = 3/791) in severity. Hypertension led to discontinuation of the study drug in 2 patients, both with a moderate case. In one of these patients, the hypertension was related to study medication: it began 28 days after the third dose of eptinezumab 100 mg and occurred intermittently for 59 days before the drug was discontinued; the hypertension was noted as resolving at the time of discontinuation. In the other patient, the hypertension was unrelated to the study drug; the event began 57 days after the first dose of eptinezumab 30 mg and continued for 3 days before study drug discontinuation.

#### Gastrointestinal events

Gastrointestinal events were reported for 221/2076 (10.6%) patients in the eptinezumab group and 68/791 (8.6%) patients in the placebo group. These events were considered treatment-related in 62/2076 (3.0%) and 18/791 (2.3%) patients, respectively. Nausea occurred in 69/2076 (3.3%) patients who received eptinezumab and 26/791 (3.3%) patients who received placebo, with 39 and 8 of these events, respectively, considered related to the study drug. Vomiting was reported for 27/2076 (1.3%) eptinezumab recipients and 9/791 (1.1%) placebo recipients, with 11 and 3 events, respectively, considered related to study medication. Most occurrences of nausea and vomiting were of mild or moderate severity; 1 nausea event and 1 vomiting event were severe. Constipation of mild or moderate severity was reported in 19/2076 (0.9%) patients treated with eptinezumab and 4/791 (0.5%) patients who received placebo. Of these events, 7/2076 (eptinezumab group) and 1/791 (placebo group) were considered related to treatment. One patient (eptinezumab 100-mg group) experienced severe constipation following a uterine suspension procedure for a retroverted uterus and uterine prolapse; however, this event was considered by the investigator as not related to study treatment.

Diarrhea was experienced by 30/2076 (1.4%) patients who received eptinezumab and 6/791 (0.8%) who received placebo. All diarrhea events were mild or moderate in severity, and 9 were considered related to study treatment (eptinezumab, *n* = 5; placebo, *n* = 4).

#### Hypersensitivity reactions

TEAEs were coded to the MedDRA preferred term of hypersensitivity (under system organ class of immune system disorders) if they occurred during study-drug infusion, led to a specific clinical action by the investigator, and were determined by the investigator to be a possible allergic response or infusion reaction. TEAEs were coded to hypersensitivity for 23/2076 (1.1%) patients treated with eptinezumab and no patients in the placebo group; associated symptoms with hypersensitivity included nasal congestion, throat discomfort (scratchiness, tightness), itching, rash, hives, watery eyes, flushing, dizziness, nausea, vomiting, rapid heartbeat, localized swelling or burning sensation, sneezing, coughing, and wheezing. In the multiple-dose studies, most events occurred following the first or second infusion. All events coded to hypersensitivity were mild or moderate in severity and were managed effectively by study-site personnel with standard medical treatment or observation alone (without treatment). All events resolved, usually within 1 day. One serious event (anaphylactic reaction of moderate [grade 2] severity) was reported and has been described above.

When considering the various TEAE terms that may indicate hypersensitivity reactions (hypersensitivity, urticaria, flushing/hot flush, rash, and pruritus), hypersensitivity reactions in the two pivotal placebo-controlled PROMISE studies occurred in at least 2% of patients in the eptinezumab 100-mg or 300-mg groups with an incidence of at least 2 percentage points greater in either of these groups than in the placebo group.

## Discussion

Pooled safety data from 5 clinical studies [12–16] have established that eptinezumab administered every 12 weeks by IV administration for the prevention of migraine in adults has a favorable safety and tolerability profile. The overall incidences of TEAEs among patients who received eptinezumab 100 mg or eptinezumab 300 mg (approved dose levels in the US) were similar to the TEAE incidence among patients who received placebo. Most TEAEs in the individual treatment cohorts were mild or moderate in severity. Overall, only 3 patients who received eptinezumab experienced a severe event that was considered related to study treatment. Throughout the clinical trial program for eptinezumab, there was a low incidence of upper respiratory, gastrointestinal, and drug hypersensitivity TEAEs that were severe, serious, or considered related to treatment by the investigator.

Although migraine has been well documented as a disabling condition that places a large burden on patients, families, the workplace, and the health care system, preventive treatments for migraine have been significantly underutilized [17, 18]. This is largely due to the marginal efficacy and poor tolerability of older preventive treatments. As demonstrated by this pooled analysis of data for more than 2000 eptinezumab-treated patients, representing nearly 5000 infusions, treatment generally was well tolerated; there were few severe or serious TEAEs, and the rates of treatment interruption and discontinuation were low. Moreover, even fewer TEAEs were considered to be related to treatment. These data combined with the published efficacy benefits of eptinezumab for the preventive treatment of EM and CM [14, 15] indicate that eptinezumab 100-mg and 300-mg doses can be safely administered to patients and confirm the clinical utility of this therapy for migraine prevention.

In a 2020 meta-analysis of data from 11 randomized, double-blind, placebo-controlled studies of CGRP monoclonal antibodies in patients with EM, injection-site pain was the only AE that occurred more frequently among patients treated with a CGRP monoclonal antibody than among those who received placebo (odds ratio [95% confidence interval], 1.44 [1.13, 1.84]; *p* = 0.004) [6]. Rates of injection-site pain in pivotal studies ranged from 1.7% to 29.8% (erenumab, 1.7%–6.0%; fremanezumab, 29.8%; galcanezumab, 6.5%–9.0%) [19–24]. In contrast, injection-site pain was uncommon in the current pooled analysis of IV eptinezumab, affecting only 4/2076 (0.2%) patients who received this drug and 1/791 (0.1%) patients who received placebo. Other infusion-site events occurred infrequently and in similar proportions of the eptinezumab and placebo groups. Extravasation, the most common of these, and pain are well-accepted and inherent potential risks associated with the method of administration. It is likely that the unique route of administration for eptinezumab (IV), as opposed to the subcutaneous delivery of other antibody treatments, underlies the large difference in these rates.

Upper respiratory AEs, such as nasopharyngitis and upper respiratory tract infection, have been reported in clinical trials of CGRP monoclonal antibodies. In a meta-analysis of EM studies, the frequency of these events in antibody-treated patients (nasopharyngitis, 6.3%; upper respiratory tract infection, 6.9%) was comparable to that of placebo patients (nasopharyngitis, 6.7%; upper respiratory tract infection, 5.9%) [6]. However, other recent meta-analyses have suggested a possible association between upper respiratory infections and erenumab [25] and galcanezumab [26, 27]. In the present pooled analysis of eptinezumab studies, the rates of nasopharyngitis (eptinezumab, 6.7%; placebo, 5.2%) and upper respiratory tract infection (eptinezumab, 7.6%; placebo, 6.1%) were similar to the EM meta-analysis [6]. Nasopharyngitis occurred in at least 2% of patients in the eptinezumab 300-mg group and with an incidence of at least 2% greater than in the placebo group. However, in most patients, nasopharyngitis was considered unrelated to study treatment. Most nasopharyngitis events occurred following the first infusion, and the rates decreased markedly for subsequent doses.

The overall low incidence of cardiovascular AEs in this analysis is consistent with the published literature for anti-CGRP therapies [28–30]. However, as in the current studies, previous investigations of other anti-CGRP monoclonal antibodies excluded patients with evidence of significant cardiovascular disease; therefore, the long-term cardiovascular safety in high-risk patients, including those with significant cardiovascular history or risk factors, remains unknown. The eptinezumab development program generally excluded patients with uncontrolled or newly diagnosed primary hypertension (systolic blood pressure > 139 or diastolic blood pressure > 89) from study participation, and the incidence of hypertension in this pooled analysis was low (< 1%). As it has been estimated that 28% to 34% of patients with migraine in the community setting have hypertension [31], further evaluation of treatments in this subset of patients is warranted. New-onset hypertension or worsening of preexisting hypertension has been associated with the CGRP receptor antibody erenumab, typically within 7 days following administration of the first dose [7].

Increased risk of gastrointestinal events, namely constipation, have been reported in clinical studies of erenumab, with post-marketing studies indicating a possible increased risk of constipation with serious complications that resulted in a warning update to the prescribing information [7, 32]. Treatment-related gastrointestinal events were reported for 3.0% and 2.3% of patients in the pooled eptinezumab and placebo groups, respectively. Most of these events were mild or moderate in severity, and none led to discontinuation of the study drug.

Events coded to hypersensitivity were uncommon, occurring in 1.1% of patients in the pooled eptinezumab population. No events coded to hypersensitivity were serious. In general, a conservative medical approach was taken: the standard recommendation was discontinuation of the study drug with no rechallenge. All events were resolved, usually within 1 day. However, if additional TEAE terms that could indicate hypersensitivity are considered (e.g., urticaria, flushing/hot flush, rash, and pruritus), hypersensitivity reactions in the two pivotal placebo-controlled PROMISE studies occurred in at least 2% of patients in the eptinezumab 100-mg or 300-mg group and with an incidence of at least 2 percentage points greater in either of these groups than in the placebo group.

This pooled analysis adds to the growing body of evidence supporting the safety and tolerability of eptinezumab for the preventive treatment of migraine [6, 33–35]. However, there are noteworthy limitations that must be considered when making clinical inferences from these data. First, the patients in these studies may not completely represent individuals considered candidates for preventive treatment. In particular, patients with atherosclerosis, cardiomyopathy, coronary artery disease, serious heart rhythm abnormalities, cerebrovascular disease, diabetes, Raynaud’s syndrome, or uncontrolled or newly diagnosed primary hypertension were generally excluded from participation. Thus, the safety and tolerability of eptinezumab described herein may not be applicable to patients with these underlying medical conditions. In addition, these studies were performed only in adults; therefore, at this time eptinezumab is not recommended for the treatment of children or adolescents. Across studies, there were low numbers of enrollment for non-Caucasians and men. Another limitation is differences in study design: not all treatment arms are equally represented (e.g., eptinezumab 1000 mg was administered only as a single dose; 30 mg, 100 mg, 300 mg, and placebo, up to 4 doses). Finally, the pooled safety population was too small to assess for very rare events, but events that occurred in 0.015% of infusions would have had a > 50% chance of being seen among the nearly 5000 infusions covered by this analysis. As such, data from post-marketing studies will be necessary to fully confirm the safety profile of eptinezumab in the real-world clinical environment.

## Conclusions

In this integrated analysis of more than 2000 eptinezumab-treated adults with EM or CM, the IV administration of eptinezumab every 12 weeks, for up to 4 doses, was well tolerated and had an acceptable safety profile. Combined with the previously published efficacy results, the collective findings demonstrate the clinical benefit and safety of eptinezumab as a preventive treatment in patients with EM or CM.

## Supplementary Information

**Additional file 1.**

## Data Availability

The data reported in this manuscript are part of an ongoing, global sponsor-led clinical development and registration program. Deidentified participant data are not available for legal and ethical reasons.

## Abbreviations

AE: Adverse event
BMI: Body mass index
CGRP: Calcitonin gene-related peptide
CM: Chronic migraine
EM: Episodic migraine
IV: Intravenous
MedDRA: Medical Dictionary for Regulatory Activities
NA: Not applicable
SAE: Serious adverse event
SD: Standard deviation
TEAE: Treatment-emergent adverse event
