# Correction to: Safety and tolerability of eptinezumab in patients with migraine: a pooled analysis of 5 clinical trials

**DOI:** 10.1186/s10194-021-01253-3

**Published:** 2021-05-25

**Authors:** Timothy R. Smith, Egilius L. H. Spierings, Roger Cady, Joe Hirman, Barbara Schaeffler, Vivienne Shen, Bjørn Sperling, Thomas Brevig, Mette Krog Josiassen, Elizabeth Brunner, Loan Honeywell, Lahar Mehta

**Affiliations:** 1 StudyMetrix Research, LLC, 3862 Mexico Road, St. Peters, MO 63303 USA; 2 Medvadis Research Corporation, Boston PainCare, Waltham, MA USA; 3 Lundbeck La Jolla Research Center, San Diego, CA USA; 4 Pacific Northwest Statistical Consulting, Inc, Woodinville, WA USA; 5 Lundbeck Seattle BioPharmaceuticals, Inc, Seattle, WA USA; 6 Lundbeck LLC, Deerfield, IL USA; 7 H. Lundbeck A/S, Copenhagen, Denmark

**Correction to: J Headache Pain 22, 16 (2021)**

**https://doi.org/10.1186/s10194-021-01227-5**

Following the publication of the original article [1], the authors notified us of a few data points that require revision (marked in red below):

Incorrect:

- Nearly one-third (32.4%) of all patients were classified as obese (BMI ≥30 kg/m2), and nearly half (48.9%) had at least 1 cardiovascular risk factor at baseline.
- “Blood pressure increased” was noted for 39/2076 (1.9%) patients who received eptinezumab and 14/791 (1.8%) patients who received placebo, …

Correct:

- Nearly one-third (32.4%) of all patients were classified as obese (BMI ≥30 kg/m^2^), and nearly half (48.8%) had at least 1 cardiovascular risk factor at baseline.
- “Blood pressure increased” was noted for 14/2076 (0.7%) patients who received eptinezumab and 5/791 (0.6%) patients who received placebo, …

The original article has been corrected.
