# Supplementary material for: Safety and tolerability of eptinezumab in patients with migraine: a pooled analysis of 5 clinical trials
Source: J Headache Pain. 2021 Mar 30;22(1):16. doi: 10.1186/s10194-021-01227-5 (PMC8008612; doi:10.1186/s10194-021-01227-5)
Supplement: Supplementary file 1 — Additional file 1. [file 10194_2021_1227_MOESM1_ESM.pdf]

Supplemental Table 1. Summary and relatedness of severe (grade 3) treatment-emergent adverse events (TEAEs) occurring in ≥2 patients in the pooled safety population

	Eptinezumab						Placebo
Patients, n (%)	**1000 mg (n = 81)**	**300 mg (n = 823)**	**100 mg (n = 701)**	**30 mg (n = 341)**	**10 mg (n = 130)**	**All (N = 2076)**	**(N = 791)**
**Any severe TEAE**	3 (3.7)	29 (3.5)	13 (1.9)	8 (2.3)	1 (0.8)	54 (2.6)	19 (2.4)
Related	1 (1.2)	1 (0.1)	0	1 (0.3)	0	3 (0.1)	1 (0.1)
Not related	2 (2.5)	28 (3.4)	13 (1.9)	7 (2.1)	1 (0.8)	51 (2.5)	18 (2.3)
**Cholelithiasis**	0	1 (0.1)	2 (0.3)	0	0	3 (0.1)	0
Related	0	0	0	0	0	0	0
Not related	0	1 (0.1)	2 (0.3)	0	0	3 (0.1)	0
**Ankle fracture**	0	0	0	0	0	0	2 (0.3)
Related	0	0	0	0	0	0	0
Not related	0	0	0	0	0	0	2 (0.3)
**Electrocardiogram T-wave inversion**	0	2 (0.2)	0	0	0	2 (<0.1)	0
Related	0	0	0	0	0	0	0
Not related	0	2 (0.2)	0	0	0	2 (<0.1)	0
**Musculoskeletal pain**	0	0	0	0	0	0	2 (0.3)
Related	0	0	0	0	0	0	0
Not related	0	0	0	0	0	0	2 (0.3)
**Uterine leiomyoma**	0	2 (0.2)	1 (0.1)	0	0	3 (0.1)	0
Related	0	0	0	0	0	0	0
Not related	0	2 (0.2)	1 (0.1)	0	0	3 (0.1)	0
**Migraine**	1 (1.2)	3 (0.4)	1 (0.1)	2 (0.6)	0	7 (0.3)	5 (0.6)
Related	0	0	0	0	0	0	1 (0.1)
Not related	1 (1.2)	3 (0.4)	1 (0.1)	2 (0.6)	0	7 (0.3)	4 (0.5)
**Syncope**	0	0	1 (0.1)	0	0	1 (<0.1)	2 (0.3)
Related	0	0	0	0	0	0	0
Not related	0	0	1 (0.1)	0	0	1 (<0.1)	2 (0.3)

Relationship of TEAE to study drug was determined by the investigator.
